# Potential Consequences of Abandonment in Preschool-Age: Neuropsychological Findings in Institutionalized Children

**DOI:** 10.3233/BEN-2012-110205

**Published:** 2012-02-15

**Authors:** Juan F. Cardona, Facundo Manes, Josefina Escobar, Jéssica López, Agustín Ibáñez

**Affiliations:** ^1^Laboratory of Experimental Psychology and NeuroscienceInstitute of Cognitive NeurologyFavaloro UniversityBuenos AiresArgentina; ^2^Psychology SchoolPontificia Universidad Católica de ChileSantiagoChile; ^3^Laboratory of Cognitive and Social NeuroscienceUniversidad Diego PortalesSantiagoChile; ^4^National Scientific and Technical Research CouncilBuenos AiresArgentina; ^5^Faculty of PsychologyUniversidad Abierta InteramericanaBuenos AiresArgentina; ^6^Psychology SchoolUniversidad SurcolombianaNeivaColombia; ^7^Universidad Autónoma del CaribeBarranquillaColombia

**Keywords:** Abandonment, institutionalization, plasticity, preschool-age, attention, memory, executive functions

## Abstract

*Objective:* Several longitudinal studies had shown that early deprivation and institutionalization during the first six months of life affects the emotional, cognitive, social and neurophysiologic development. Nevertheless, our understanding of possible similar effects of delayed institutionalization, in preschool-age remains unclear to this day. The goal of this study is to evaluate the cognitive performance of institutionalized children with history of preschool-age physical abandonment.

*Method:* 18 male institutionalized children with history of abandonment during the preschool-age (2–5 years old) and comparison group matched by age, handedness, gender, educational and socioeconomic level were tested on multiple tasks of attention, memory and executive functions.

*Results:* We found a cognitive impairment in the institutionalized children in several measures of attention, memory and executive functions. This is the first report of cognitive impairment related to late abandonment and institutionalization effects (after 2 years old), extending the already known effects on early institutionalization.

*Conclusions:* This preliminary study suggests that environmental factors including abandonment and institutional care, can affect not only the infancy period, but also the preschool period providing new insights into our understanding of neurocognitive development.

## 1. Introduction

The negative impact of parental deprivation and institutional rearing in childhood has been a growing area of scientific and clinical interest since the mid-20th century. Pioneering work of Provence and Lipton [1] has shown that maternal deprivation and institutional rearing during the first years of live produces a negative effect and triggers development abnormalities. Several reports has suggested that deficitary parental care in this sensitive period is associated with physiological dysregulation of the developing human brain [2,3].

Most of the recent information we have concerning the impact of child neglect and institutionalization on neurodevelopment has been after 1989, with the devastating effect of the Romania institutionalization project,

ISSN 0953-4180/12/$27.50 © 2012 – IOS Press and the authors. All rights reserved and the consequent development of The Bucharest Early Intervention Project (BEIP). Now, the neurocognitive effects of the early institutionalization are widely recognized in children [4–6].

Several longitudinal studies had shown that early institutionalization including the first six months of life affects dramatically the emotional, cognitive, social and neurophysiologic development [7–11]. Numerous investigations [12–16] have shown long-term impairments on the emotional, cognitive and social development in children with history of early institutionalization. Vorria et al. [17], found a deficient performance on cognitive development, attachment, emotional processing as well as abnormal behavior in 61 institutionalized children (during the two first years of life) compared to non institutionalized controls. Other studies reported similar results [4,18]. Nevertheless, our understanding of possible similar effects of delayed institutionalization in preschool-age is not well known. The goal of this study is to evaluate the cognitive performance of children abandoned and institutionalized after the second year of life.

### 1.1. Attention, working memory and executive function in abandoned children

One recent study [5] focused on cognitive outcomes of adopted children with a history of institutional care at 8–10 years old. Neuropsychological assessment included memory, learning, attention, and executive function. Three groups were considered: post-institutionalized adopted children (infants adopted after 12 months of age, with 75% of their lives under institutional care), early adopted children (defined as children adopted prior to 8 months of age, with 2 months or less under institutional care) and a control group. Post-institutionalized children obtained lower scores in visual memory and attention, as well as impairments on visually mediated learning and inhibitory control, compared with the other two groups.

Bos et al. [19] using the Cambridge Neuropsychological Test and Automated Battery (CANTAB), reported impairments in visual memory, spatial working memory and executive functions in children whit history of early institutional care. Symptoms of attention deficit and hyperactivity disorder (ADHD) related to history of institutionalization has been reported elsewhere [20]. In addition, Vorria et al. [17], and Roy et al. [21], reported deficits in attention and hyperactivity behavior in institutionalized children (see also Kreppner et al. [8]).

### 1.2. Institutionalization onset

Infancy is recognized as the period between the second postnatal month and two years, previous to the mid-childhood. In humans the period is extremely sensitive to maternal investment mother dependence for survival, and is characterized by rapid neural growth [22]. Several reports cited above show that the early institutionalization induces dramatic biological and cognitive disorders, especially when the institutionalization period begins during the first year of life. This is consistent with the neurodevelopment approaches which suggest the existence of sensitive or critical periods of growth [23]. These critical periods are sensitive to specific environmental influences which are required for the normal development of anatomical and functional properties of the brain [24,25].

Studies considering the effects of institutionalization after the infancy are scarce. Recently, late plasticity effects and a life span developmental process have been established [26,27]. Those findings indicate that adverse early experience can have long-term impact on brain development. In this sense, similar cognitive deficits are predicted in children institutionalized after the infancy period.

In Latin America, multiple factors affect the normal development of numerous children [28–30]. In Colombia, the Familiar Welfare Colombian Institute [31] reported 4500 institutionalized children during the 2006. This phenomenon is a consequence of a multitude of problems such as physical maltreatment, childhood neglect, parental inadequacy, parent's death. Nevertheless, one of the most influential causes is the extremely poor socioeconomic conditions of the family.

In this pilot study, we examined cognitive functioning using a battery of neuropsychological tests measuring attention, learning and memory, visual-spatial processing, executive functions and psychomotor functioning, in institutionalized infants with a history of abandonment in preschool-age and sociodemographically similar comparison children who had not been emotionally and/or physically deprived.

## 2. Methods

### 2.1. Participants

#### 2.1.1. Preschool-age physically abandoned children (PPAC)

Table 1 shows sample characteristics of eighteen children from the south of Colombia, with a history

Table 1

Descriptive characteristics of the two groups studied

| | Institutionalized Children $N = 18$ | | Comparison Group $N = 18$ | | T value or Chi-square value | $p$ |
|---|---|---|---|---|---|---|
| | M | SD | M | SD | | |
| Age (years) | 11.5 | 2.52 | 11.5 | 2.52 | $X^2_{(8)} = 0.53$ | $> 0.05$ |
| Gender (male)* | 18 | – | 18 | – | – | – |
| Education (years) | 5.28 | 2.30 | 5.61 | 2.50 | −0.41 | 0.68 |
| Handedness (L/R)* | (1/17) | – | (1/17) | – | – | – |
| Abandonment (age) | 3.50 | 1.50 | – | – | – | – |
| Institutionalization (years) | 7.56 | 2.68 | – | – | – | – |
| CDI† | 8.40 | 3.25 | 6.20 | 1.22 | 3.02 | 0.006 |

†A cut-off value of 11 was established for distinguishing depressed children from non-depressed children [32].
*In both, *gender* and *handedness* no statistical comparison was performed because SD and group differences were absent.
CDI: Children's Depression Inventory.

of abandonment after the second year of life ($M = 3.5$; SD = 1.52); reasons for placement were extremely poverty conditions (i.e., inability to satisfy basic needs in food, clothing, shelter, and health) with no indicators of physical (e.g. absence of cutaneous and mucosal, dental, osteo-articular, organic internal lesions or intoxication) or sexual abuse (e.g. absence of genital or rectal lesions, sexual transmitted diseases); with a history of institutionalization greater than three years ($M = 7.56$; SD = 2.68); and 7 to 15 years old ($M = 11.5$; SD = 2.52). It should be noted that we couldn't rule out with total certainty any antecedent of physical or sexual abuse because those indicators tend to be underdiagnosed [32]. See discussion regarding this issue. All children were in school at the time of the study ($M = 5.28$; SD = 2.30), with normal weight/size and affiliated to a health care system. Children without a history of developmental or behavioral disorders were included in this study, in order to discard any possible confounder.

### 2.1.2. *Comparison Group* (*CG*)

Eighteen healthy children were recruited at the same school. They were living with their biological parents, and had no history of institutional care. All the children in comparison group come from a low socioeconomic environment, according to the guidelines of the Colombian State belonged to the stratum 1. In Colombia, social status is classified through socio-economic levels called stratums; the lowest corresponds to level 1 defined by living in poor quality housing, living in a place that lacks utilities, or living in overcrowded, defined as three or more persons per room. This groups was matched to PPAC group in terms of age ($M = 11.5$; SD = 2.52), educational level ($M = 5.61$; SD = 2.50) and handedness. No history of physical or psychological abuse was present in the CG, as confirmed by a social worker.

In order to test the possible mood disorders present in both groups, the Children's Depression Inventory (CDI) [33] was included in the neuropsychological assessment. No children coming from both groups which presented scores indicative of depression were included in the present study (See Table 1). Since that CDI scores were significant different between groups (Table 1), we performed an ANCOVA in order to establish if CDI have an impact on neuropsychological groups' differences. When CDI was introduced as covariate in the between groups comparisons, all neuropsychological differences were still significant. Based on this result we concluded that CDI have not effect in the between groups differences.

Both groups included only subjects with normal IQ assessed whit the Kaufman Brief Intelligence Test [34] (mean IQ scores over 85 points for composite, fluid and crystallized intelligence). Nevertheless, IQ individual scores were not computed and we are unavailable to recover those scores. Because of that, we did not report IQ distribution.

All participants signed a voluntary consent form in accordance with the Declaration of Helsinki, in addition to the approval granted by the ethical committee of the institution. The ethical committee of the orphanage approved this research and the director of the institution signed the voluntary consent together with the institutionalized children. All parents and children from the comparison group signed the voluntary consent in agreements with the declaration of Helsinki.

For both groups the following exclusion criteria were considered: mental deficiency, neurologic antecedents (stroke, epilepsy or lesions) or psychiatric antecedents

(history of hospitalization, diagnosis of developmental); substance abuse, AIDS, malnutrition, physical or sensorial limitations. All this information was obtained simultaneously by the parents and by the clinical history.

### 2.2. Characteristics of orphanage

The orphanage is an institution of the National Family Welfare System in Colombia, which welcomes children between 3 and 14 year, without severe mental or physical disabilities (e.g., Down syndrome or cerebral palsy), in situation of social risk-shifting, abuse or abandon, from a marginal sector of the country. This institution has a care capacity (including medical care, nutrition, sanitation) and free elementary school up to 200 children.

The institution includes medical personnel, clinical psychologists, special teachers, social workers, nutritionist and caregivers who are present all the time. In addition, voluntary professionals from the community (e.g., teachers, specialist for physical education, psychology and caregivers), support the work at the orphanage. Children are grouped homogeneously with respect to age, and each group has its daily prescribed schedule of group sleeping, feeding, and indoor/outdoor plays times. In addition, the institution has a five class rooms, two playgrounds, food preparation room, and a dining room for all the infants.

### 2.3. Neuropsychological battery

The NEUROPSI Attention and Memory Battery [35] was administered to participants in order to evaluate orientation, attention, concentration, executive functions, working memory, verbal memory and visual memory (immediately and delayed). This battery combines a set of international validated measures and has been validated and standardized for Spanish populations. Subtests were grouped in order to obtain a global index of attention, executive function and memory, as well as a global score of attention, executive functions and memory. Raw scores were transformed to normalized t-scores based on age, according to scoring procedure of the neuropsychological instrument. Details of the NEUROPSI battery can be found in the supplementary data.

### 2.4. Data analysis

Different neuropsychological subtests from PPAC and CG were compared using independent samples t-tests since both groups were homogeneous regarding age, educational level, socioeconomic level, and handedness. For all analysis, a level of $p = 0.05$ was considered for statistical significance. Means and SD were calculated for confidence intervals of 95%, winch represents $\pm$ 2 SD. The global scores were correlated with age in both groups using Spearman's rank correlations.

## 3. Results

Table 2 presents the overall results for both groups. PPAC obtained significantly poorer results compared to CG on all cognitive scores.

Temporal and spatial orientation was impaired in PPAC group compared with CG, however, person orientation was preserved in both groups. In the domains of attention and concentration, PPAC group presented an impaired performance in those measures (i.e., digit forward span, mental control and spatial forward span).

The PPAC group performed more poorly than CG on executive functions subdomains, mainly in abstraction or concept formation ability (i.e., category formation test), and inhibitory tasks administered, including the Stroop color and word test color/word, a stimulus-response reversal task, and a motor programming task. By contrast, no significant differences between groups were found on verbal fluency tasks (e.g., phonological and semantic).

The immediately memory scores from the PPAC group presented also a deficit compared to CG in several subtest, including tasks that require recall of semantically unrelated material (i.e., word list learning); retention of information test (i.e., logical memory retained) and measures of visuospatial function (i.e., Rey-Osterreith complex figure and face recognition test).

In addition, PPAC performed poorly than CG on tasks that require recognition of previously presented items (i.e., word list recall and cued recall, verbal paired associated; and logical memory)

Finally, working memory task was impaired in PPAC in comparison to CG in the digit backward span subtest.

### 3.1. Correlations

In the CG, age correlated with attention and executive functions total score ($r = 0.6$; $p < 0.05$); and whit memory total score ($r = 0.4$; $p < 0.05$). In the PPAC, similar to CG, age correlated with attention and executive functions total score ($r = 0.6$; $p < 0.05$); and with memory total score ($r = 0.8$; $p < 0.05$). No other significant correlations were found.

Table 2
Neuropsychological test scores of institutionalized children and comparison group

| Cognitive domain | Institutionalized children M | Institutionalized children SD | Comparison children M | Comparison children SD | Analysis df | Analysis t | Analysis p |
|---|---|---|---|---|---|---|---|
| Orientation | | | | | | | |
| Time (4) | 3.17 | 1.15 | 3.94 | 0.23 | 18 | −2.810 | 0.008 |
| Place (2) | 1.44 | 0.70 | 1.89 | 0.32 | 23 | −2.432 | 0.01 |
| Person (1) | 1.00 | 0.00 | 1.00 | 0.00 | – | – | 0.2 |
| Attention and concentration | | | | | | | |
| Digit forward span (9) | 4.44 | 1.04 | 6.11 | 0.58 | 26 | −5.924 | < 0.001 |
| Digit detection (10) | 8.72 | 1.67 | 9.39 | 0.77 | 24 | −1.533 | 0.12 |
| Mental control (3) | 1.33 | 1.37 | 2.94 | 0.24 | 18 | −4.910 | < 0.001 |
| Spatial forward span (9) | 5.06 | 0.80 | 6.11 | 1.13 | 34 | −3.228 | 0.002 |
| Visual search (24) | 13.56 | 5.11 | 16.44 | 3.99 | 34 | −1.890 | 0.05 |
| Executive functions | | | | | | | |
| Category formation test (25) | 9.22 | 3.19 | 17.94 | 4.18 | 34 | −7.037 | < 0.001 |
| Semantic verbal fluency | 16.61 | 4.90 | 18.83 | 3.05 | 34 | −1.633 | 0.10 |
| Phonological verbal fluency | 9.56 | 4.50 | 10.39 | 3.24 | 34 | −0.638 | 0.52 |
| Design fluency (35) | 10.28 | 4.64 | 11.78 | 4.88 | 34 | −0.945 | 0.34 |
| Motor functions (20) | 17.89 | 2.27 | 19.33 | 0.90 | 22 | −2.505 | 0.01 |
| Stroop (time) | 2.11 | 1.28 | 2.44 | 0.98 | 34 | −0.877 | 0.03 |
| Stroop (correct) (36) | 1.44 | 0.86 | 2.17 | 1.09 | 32 | −2.201 | 0.03 |
| Working memory | | | | | | | |
| Digit backward span (8) | 3.00 | 1.03 | 4.28 | 1.02 | 34 | −3.746 | < 0.01 |
| Spatial backward span (8) | 4.72 | 1.02 | 5.28 | 1.48 | 30 | −1.308 | 0.186 |
| Immediate memory | | | | | | | |
| Word list (12) | 5.89 | 1.32 | 7.00 | 1.41 | 34 | −2.434 | 0.02 |
| Verbal paired associated (12) | 7.44 | 2.45 | 8.44 | 1.38 | 34 | −1.506 | 0.13 |
| Logical memory (16) | 8.16 | 2.30 | 12.06 | 1.66 | 34 | −5.803 | < 0.001 |
| Rey-Osterreith complex figure (36) | 23.16 | 9.15 | 31.27 | 3.23 | 21 | −3.548 | < 0.01 |
| Faces (4) | 2.72 | 1.01 | 3.72 | 0.67 | 34 | −3.483 | 0.001 |
| Delayed memory | | | | | | | |
| Word list (free recall) (12) | 7.00 | 1.32 | 8.39 | 1.91 | 30 | −2.529 | 0.01 |
| Word list (cued recall) (12) | 6.83 | 2.09 | 8.61 | 1.82 | 34 | −2.719 | 0.01 |
| Word list (recognition) (12) | 10.39 | 1.75 | 11.06 | 0.99 | 26 | −1.623 | 0.15 |
| Verbal paired associates (12) | 8.89 | 2.27 | 10.44 | 1.82 | 34 | −2.266 | 0.03 |
| Logical memory (16) | 8.17 | 2.03 | 11.77 | 1.63 | 34 | −5.875 | < 0.001 |
| Rey-Osterreith complex figure (36) | 16.66 | 6.33 | 20.98 | 3.82 | 27 | −2.471 | 0.03 |
| Faces (2) | 1.61 | 0.60 | 1.67 | 0.59 | 34 | 0.338 | 0.76 |
| Attention and executive functions total score | 73.44 | 15.90 | 110.83 | 11.26 | 34 | −8.139 | < 0.001 |
| Memory total score | 82.05 | 19.89 | 114.39 | 8.78 | 23 | −6.308 | < 0.001 |
| Attention, executive functions and memory total score | 74.78 | 17.23 | 115.55 | 9.78 | 26 | −8.732 | < 0.001 |

Numbers within parenthesis are indicative of maximum score.

## 4. Discussion

The present study examined the profile of neuropsychological measures of attention, memory and executive functions in institutionalized participants with history of abandonment during the preschool-age (2–5 years old) and age, education, socioeconomic status, handedness and gender-matched controls. Results indicate a poor performance in the PPAC group compared to CG in virtually all subtests of attention, memory and executive functions. Our results support an extended view of early life stress (ELS), indexed by adverse care and lack of an adequate socio-emotional attachment, as having an impact on neurocognitive development. Probably, the stress induced by institutionalization can affects the neurobiology of stress and the neurobiology of rapid threat appraisal and response [36]. The consequences of our results are twofold. At theoretical level, our data suggest that late institutionalization can trigger similar effects as ELS, stressing the role of more delayed critical periods of development. At practical level, our result highlights the importance of intervention program developments for late institutionalized children.

This report confirms previous studies performed with early institutionalized children [5,18–20,37]. These deficits in the neuropsychological assessment hint at a possible involvement of abnormal processing of prefrontal cortex and medial temporal areas, as hippocampus, in a similar vein with early institutionalization previous studies [5,12,13,36,38].

However, the PPAC group presented a normal performance in some tasks which evaluate verbal (phonological and semantic fluency test) and visual fluency functions (design fluency test). Similar results have been reported in other studies [5,39]. Presumably these functions are less vulnerable to institutionalization experiences. It can be speculated that delayed maturation of select aspects of frontal circuitry, and perhaps reduced functional connectivity of frontal cortex with other cortical and subcortical regions [5] would explain those differences.

Attention, executive functions and memory were correlated with age in both groups, suggesting that those cognitive functions are sensitive to age effects. This suggests that cognitive evolution can be considered similar in both groups even with a delayed and impaired performance in PPAC. Similar results have been reported in other studies [5,13,19], that describe alterations in attention, memory and executive functions affected by the age of participants.

To our knowledge, this is the first report of cognitive effects of abandonment in preschool-age. Moreover, this is the first study which shows similar cognitive impairments of attention, memory and executive functions to those already reported in institutionalized children exposed to early parental deprivation. Our results suggest that deprivation/ disruption of parental care in the preschool-age still constitute an important risk factor in the development of attention, memory and executive functions. Furthermore, institutional rearing seems to be an adverse caregiving environment.

Several pathways suggest that parental care plays an important role in the development of neural system in early and late infancy. For instance, is has been shown that: (a) parents trained to control behavior problems increases the secure attachment behavior for preschooler; (b) the adverse care from institutionalization and other caregivers increases the risk of psychopathology; (c) children exhibits larger cortisol stress responses when parent are rated as low in sensitivity; and (d) foster parents' who provide supportive care improves the behavioral functioning of preschoolers (all reviewed in Loman & Gunnar [36]). At the same time, the absence of parent care increases the risk of aggressive and dangerous interactions with adults and peers. Thus, parental care and attachment process (physiologically indexed by the stress, the neuroendocrine and neurotransmitter systems), would have a deeper effect not only in the early, but also in the late infancy.

Our results are consistent with current views of development and neurodevelopment. The current consensus is that the parental care plays a critical role in the development of the neural systems [36]. In that sense, the nervous system has long periods of development, with myelination and synaptogenesis continuing through puberty in animals and humans. The neural reorganization (adaptative or deficitary) is not limited to the early stages of development [27]. In the last decade, several reports have evidenced continued development of the brain through late childhood, adolescence and even young adulthood [40–42]; for review see Rice and Barone [43]. At the same time, theories of development involve a lifespan perspective encompassing different stages of development [26].

Previous studies in early institutionalized children had shown that impaired executive functions are frequently accompanied with deficits in theory of mind and emotion processing [44]. At the same time, attention, memory and executive function deficits have strong effects on the emotional development [10,45]. Recently, has been proposed that basic frontal executive function is affected by and affecting the emotional, motivational and social process [46]. Complex cognitive behaviors have their basis in dynamic coalitions of neural networks related to executive as well as affective process. Thus, a relatively early impairment of executive function and memory would impair the elemental mosaics of social and emotional cognition, affecting the progressive socio-emotional development. Future studies on late institutionalized children should include evaluation of emotion processing and theory of mind measures.

### *4.1. Limitations and further assessment*

There are several limitations that make our result preliminary. The same size of our repot is small. Nevertheless, our study did include a comparison group which was precisely matched on a number of different factors. We strongly recommend that future studies be conducted on larger samples including both genders. Furthermore, we were unable to retrieve a detailed history of children prior to their institutionalization (i.e., psychological and physical abuse, witnessing partner violence). Additionally, we do not know if our findings are related to abandonment, institutionalized or a combination of both factors.

Despite these limitations, our results are consistent with previous reports [19,47,48] showing the damaging effects of physical abandonment and institutional rearing during the infancy period. Our result, extending those reports during the infancy, provides new insights into the understanding of neurocognitive development. Future studies will be needed to further examine whether the same potential effects of child neglect can be observed in abandoned, institutionalized children in foster homes.

## 5. Conclusion

This preliminary report shows a strong cognitive impairment among institutionalized children with a history of preschool-age abandonment in core process as attention, memory and executive functions.

To our knowledge, there are no previous reports of preschool abandonment effects on the cognitive development, extending the already known effect on early institutionalization. This study suggests that parental deprivation and environmental factors including institutionalization can affect not only the infancy period, but also preschool age and mid-childhood, requiring a revised model of neurocognitive development.

## Acknowledgments

The authors would like to thank the Familiar Welfare Colombian Institute, who facilitated the recruitment of institutionalized participants. This study was partially founded by CONICET and FINECO grants.

## Supplementary data

The following sections are included in the Neuropsi Attention and Memory Battery.

*Orientation*

This task comprises general information regarding subject's orientation in time, space and person and includes declarative memory for personal relevant information (e.g., date, age) (maximum score = 7 points). Subject must respond to questions regarding personal knowledge (e.g., day of the week, neighborhood, age, etc.).

*Attention and concentration*

a. Auditory/verbal

I) Digit forward span. This consisted of pairs of random number sequences that the examiner read aloud, at the rate of one per second. The subject's task was to repeat each sequence exactly as it was given. Task is stopped by the examiner after two incorrect answer (maximum score = 9 points).

II) Digit detection. This vigilance test examined the ability to sustain and focus attention. It involved the sequential presentation of digits over a period of time with instructions for the patient to tap only when the target item 5 was preceded by the item 2 (maximum score = 10 points).

III) Mental control. This task required the subject to count from 1 to 40, in 3s, within a time limit (maximum score = 3 points).

b. Visual/nonverbal

I) Spatial forward span. This consisted of a board with blocks attached in an irregular arrangement. In the test, each time the examiner tapped the blocks in a prearranged sequence, the patient attempted to copy this tapping pattern exactly. Task is stopped after two incorrect answers (maximum score = 9 points).

II) Visual search. This test required visual selectivity at fast speed on a repetitive motor response task. It consisted of rows of figures randomly interspersed with a designated target figure. The subjects were requested to cross out those figures equal to the one presented as a model. The person indicates when finished, or is stopped after 60 seconds. Two scores were obtained: total number of correct responses (maximum score = 24), and number of intrusions.

*Memory*

a. Working memory

I) Auditory/verbal: Digit backward span. This consisted of pairs of random number sequences that the examiner read aloud, at the rate of one per second, and the subject's task was to repeat each sequence in an exactly reversed order. Task is stopped after two incorrect answers (maximum score = 8).

II) Visual/nonverbal: Spatial backward span. This consisted of a board with blocks; each time the examiner tapped the blocks in a prearranged sequence, the patient attempted to copy the tapping pattern in an exactly reversed order. Task is stopped after two incorrect answers (maximum score = 9).

b. Auditory/verbal immediate and delayed recall

I) Auditory/verbal immediate and 20-minute delayed recall of word list (three learning trials of 12 words). Immediate presentation consisted of three presentations with recall of a 12-word list. Each of the 12 items belonged to one of three semantic categories (animals, fruits, or body parts). After each presentation, the subject repeated those words that he/she remembered. The total score was the average number of words repeated in the three trials (maximum score = 12). The delayed presentation provided one first free recall after 20 minutes (maximum score = 12). The second long term recall trial utilized the item categories as cues, asking the subject for items in each of the three categories (maximum Score = 12). A recognition trial was also provided, in which the examiner asked the subject to identify as many words as possible from the list from a new list of 24 words. This new list contained all the items from the original list, as well as words that were semantically associated or phonemically similar (maximum score = 12 points). In addition, intrusions, perseverations, and false positive error scores were noted in delayed trials.

II) Verbal paired associates. Twelve word pairs, four that were not readily associated (i.e., coche – payaso), four forming phonetic associations (i.e., camion–melon), and four forming semantic associations (i.e., fruta– uva). The list was read three times, with a memory trial following each reading. The words were randomized in each of the three learning trials to prevent positional learning. The total score was the average number of correct word pair repeated by the participant in the three trials (maximum score = 12). A 20-minute delayed recall was also provided (maximum score = 12). In addition, intrusions, perseverations, and errors were noted.

b. Logical memory I and II. Prose learning that allowed scores of thematic recall and factual knowledge. The examiner read two stories, stopping after each reading for an immediate free recall. In the two paragraphs, subject must recall many details as possible, in both immediately, and after delay. Each story contained 16 story units and 5 thematic units. A delayed recall trial after 20 minutes was also given. For instance, correct scores are the sum of the correctly recalled story and thematic units.

c. Visual/nonverbal immediate and delayed recall

I) Rey-Osterreith Complex Figure. Subjects were shown a card with a design to be copied. A delayed recall was also provided after 20 minutes in which subjects were asked to recall what they had drawn on the administration trial. The figure is broken down into 18 scorable elements: between 0.5 and 2.0 points are awarded for each element, depending on the accuracy, distortion, and location of its reproduction. Two points are awarded if the unit is correct and is placed properly, 1 point if the unit is correct but placed poorly, 1 point if the unit is distorted but placed correctly, 0.5 points if the unit is distorted and placed poorly, and no points if the unit is absent or not recognizable. There is a 5-minutes time limit (maximum scores = 36).

II) Faces. On the immediate trial subjects were shown two photographs with their respective names. After seeing each of them for 5 seconds, subjects were asked to repeat the names (maximum score = 4 points). On the delayed recall subjects were asked to remember the names of the persons (maximum score = 8 points) and to identify the previously shown persons from among a set of four photographs (maximum score = 2 points). In addition, false positive errors were noted.

*Executive functions*

a. Category Formation Test. This consisted of five visually presented sets, each one containing four figures of common objects. Each set was organized on the basis of different principles. On each set trial the subjects were asked to form as many categories as they could. The subject indicates when finished, or is stopped after 5 minutes (maximum score = 25).

b. Verbal fluency. This measured the number of words produced within a time limit of 1 minute and consisted of a semantic as well as a phonological trial. On the semantic trial subjects were required to generate items in a category (animals), whereas on the phonological trial they were required to generate words according to an initial letter ("P"). Total number of correct word pair, intrusions, perseverations, clusters, and switching were scored in both tests.
c. Design fluency. The subject was instructed to draw different patterns by connecting dots in a five-dot matrix using four lines, with a 3 minute time limit. Total number of correct designs, intrusions, and perseverations were quantified.
d. Stroop Test. Subjects were required to read, as fast as they could, a set of colour words printed in black ink. On the second trial, subjects were required to call out, as fast as they could, the colour names of coloured ovals. On the third trial subjects were asked to call out, as fast as they could, printed colour names when the print ink was a different colour from the name of the colour word. In the three trials, the total number of correct answers were recorded (maximum score = 36).
e. Motor functions

I) Conjugate eye movement. A pencil was shown to the subjects and they had to follow it with their eyes to the left and then to the right (maximum score = 4 points).
   II) Conflicting commands. Subjects were asked to hit the table once when the administrator hit it twice, or to hit the table twice when the administrator hit it only once. To ensure the subject had clearly understood the task, a practice trial was performed in which the administrator first hit the table once, three times in succession, and then twice, three more times (maximum score = 2 points).
   III) Go/No-Go. Subjects were told that now, when the test administrator hit the table once, they should hit it once as well, but when the examiner hit twice, they should do nothing. To ensure the subject had clearly understood the task, a practice trial was performed in which the administrator hit the table once, three times in succession, and then twice, three more times (maximum score = 2 points).
   IV) Luria's Hand Sequences. The examiner made a fist with his right hand, then extended his fingers holding his hand horizontally, and finally turned his hand by 90° with the extended fingers still pointing forward. After seeing this sequence of movements, subjects had to repeat it with their right hand exactly as shown. In a second trial the examiner repeated the sequence in an exactly reversed order with his left hand and subjects had to repeat it with their left hand exactly as shown (maximum score = 4).
   V) Alternating pattern. This task requires copying a drawing without lifting the hand from the paper. The test required alternating between peaks and blocks (maximum score = 8).
